# Methylation hallmarks on the histone tail as a linker of osmotic stress and gene transcription

**DOI:** 10.3389/fpls.2022.967607

**Published:** 2022-08-10

**Authors:** Mu Xiao, Jinbiao Wang, Fang Xu

**Affiliations:** The Key Laboratory of Plant Development and Environmental Adaptation Biology, Ministry of Education, School of Life Sciences, Shandong University, Qingdao, China

**Keywords:** osmotic stress, histone methylation, histone demethylation, gene transcription, stress memory

## Abstract

Plants dynamically manipulate their gene expression in acclimation to the challenging environment. Hereinto, the histone methylation tunes the gene transcription *via* modulation of the chromatin accessibility to transcription machinery. Osmotic stress, which is caused by water deprivation or high concentration of ions, can trigger remarkable changes in histone methylation landscape and genome-wide reprogramming of transcription. However, the dynamic regulation of genes, especially how stress-inducible genes are timely epi-regulated by histone methylation remains largely unclear. In this review, recent findings on the interaction between histone (de)methylation and osmotic stress were summarized, with emphasis on the effects on histone methylation profiles imposed by stress and how histone methylation works to optimize the performance of plants under stress.

## Introduction

Post-translational modification (PTM) is a critical regulatory mechanism of the cellular organisms, which governs all aspects of the genomic functionality from DNA (deoxyribonucleic acid) replication, to mRNA (messenger ribonucleic acid) production, and to protein turnover, enabling them to dynamically respond to developmental cues and environmental fluctuations (Ytterberg and Jensen, 2010; Hashiguchi and Komatsu, 2017; Kosová et al., 2021). In eukaryotic cells, the octameric nucleosome consists of two molecules of H2A, H2B, H3, and H4 each, which are further wrapped by 147 base pairs of DNA. The occupation of histones and modifications on the core histones primarily determines the nucleosome density and chromatin configuration, which further controls the accessibility to a repertoire of transcription factors (Jenuwein and Allis, 2001; Shahbazian and Grunstein, 2007; Deal and Henikoff, 2011; Klemm et al., 2019). Methylation on the N-terminal of histones is an important covalent modification that dictates the gene transcription programs to combat the environmental stresses (Kim et al., 2008, 2010, 2015; Yuan et al., 2013; Van Oosten et al., 2014; Asensi-Fabado et al., 2017; Wong et al., 2017). Furthermore, the methyl-groups are dynamically added to or removed from the histones, allowing precise transcriptional manipulation (Pfluger and Wagner, 2007; Kim et al., 2008; Deal and Henikoff, 2011; Xiao et al., 2016; Park et al., 2018).

Histones can be methylated at the lysine residues and the arginine residues, which are catalyzed by histone lysine methyltransferases (HKMT) and histone arginine methyltransferases (HRMT), respectively. Histone lysine methylation is specifically catalyzed by the SET-Domain Group proteins (SDGs), which can be divided into seven subfamilies including E(z), Ash, Trx, Suv, PRDM, SMYD, and SETD (Springer et al., 2003; Zhou et al., 2020). E(z), Ash, Trx, and Suv are four major subfamilies involved in the methylation of histones (Springer et al., 2003). In plants, most characterized histone lysine methylation occurs at H3K4, H3K9, H3K27 and H3K36 while the methylation on H3K79 is rare. The histone arginine methylation primarily occurs on H3R2, H3R8, H3R17, H3R26, and H4R3 (Feng et al., 2002; Niu et al., 2007; Pfluger and Wagner, 2007; Wu et al., 2009; Liu et al., 2010; Berr et al., 2011; Huang et al., 2014, 2015; Zacarias and Casas-Mollano, 2021). Histone methylation can serve either positive or negative functions in gene transcription. Methylation on H3K9, H3K27, and H4R3 marks transcriptional repression. By contrast, methylation on H3K4 and H3K36 is labelled with transcriptional activation (Yu et al., 2009; Wang et al., 2015a; Zacarias and Casas-Mollano, 2021). The histone lysine methylation and arginine methylation together build histone hallmarks that can be interpreted into transcription activation or repression. The histone methylation is a reversible process where methyl groups can be added onto specific residues by histone methyltransferases/methylases and be removed by histone demethylases, which plays vital roles in maintaining the homeostasis of histone methylation (Klose and Zhang, 2007; Liu et al., 2010). In higher plants, there are two distinct histone demethylation mechanisms: amine oxidation by LSD1 (lysine-specific demethylase1) which specifically demethylates H3K4 and hydroxylation by JmjC proteins (Jumonji domain-containing) with broader specificity (Shi et al., 2004; Tsukada et al., 2006; Williams et al., 2014; Qian et al., 2015).

**Figure 1:**
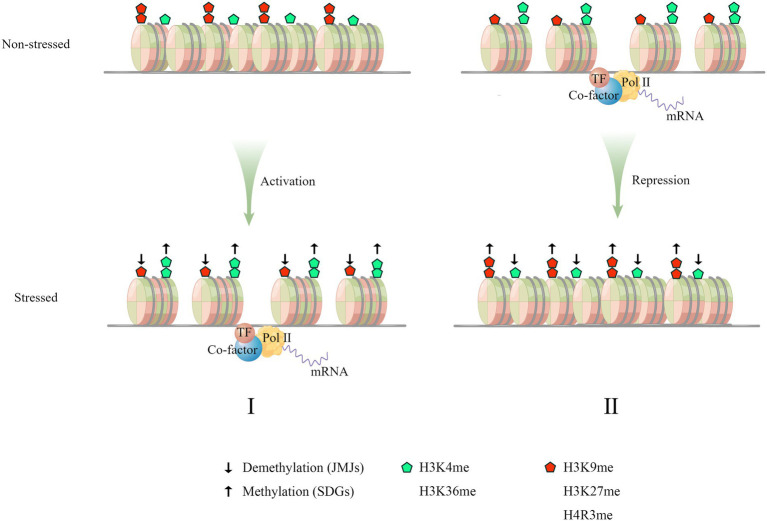
A paradigm of stress-induced histone hallmarks and gene transcription. Osmotic stress induces both transcriptional activation (I) and repression of stress-responsive genes (II). These two mechanisms collaboratively orchestrate the stress response by modulating gene transcription. (I) Under non-stressful conditions, the chromatin region of genes positively regulating stress-response is enriched in repressive histone methylations (the red pentagon) and has higher nucleosome occupancy, which decreases the chromatin accessibility to the transcription machinery. Upon stress, the chromatin region is marked with more active histone methylations (the green pentagon) and bears fewer nucleosomes, leading to increased accessibility and transcriptional activation. (II) As for the genes negatively regulating stress response, the corresponding genic loci are marked with higher active histone methylations and lower repressive histone methylations. The active transcription helps to repress inappropriate stress-response. Upon stress, the deposition of active markers is reduced while the repressive markers are deposited, which leads to transcriptional repression. This negative regulation counteracts the repression of stress response imposed by negative genes in stress signaling pathway. The depletion of histone methylations is mediated by JMJ demethylase (↓) and the increase of histone methylations is mediated by histone methylases/methyl-transferases (↑), which represents two biological processes termed histone demethylation and histone methylation, respectively. In both cases (I, II), the histone methylation and histone demethylation might be simultaneously involved.

Plants have sessile lifestyle and their vitality is stringently dependent on their growth conditions (Frolov et al., 2017). Osmotic stresses (OS), as a major common abiotic stress limiting the crop yield, are most likely to occur periodically (Ault, 2020). Drought and salinity are two representatives of osmotic stress, which are non-ionic and ionic, respectively, (Munns et al., 2010). Drought is one of the most devastating abiotic stresses in agronomy and caused by water deficit (Ault, 2020). Several other abiotic environmental factors, such as high salinity and reduced or elevated temperatures, also decrease water availability, leading to the onset of osmotic response (Verslues et al., 2006; Frolov et al., 2017). The molecular basis underpinning response to osmotic stress has been a hot topic in plant stress biology. In certain ecosystems, plants adapt to environmental stresses by different mechanism including epigenetic regulation (Granot et al., 2009; Kim et al., 2010; Balao et al., 2018). As the drought stress occurs, plants correspondingly shape their histone methylome including H3K4me2, H3K9me2/3 and H3K27me2/3. Moreover, this epigenetic mechanism seems to be associated with their living surroundings and species-specific (Granot et al., 2009). These findings evidence that plants can respond to the environment on the chromatin level. Herein we summarized recent studies on the histone methylation regulation upon osmotic stress (Table 1), discussed the underlying mechanism (Figure 1), pinpointed several questions to be addressed and postulated the perspectives of study on histone methylation.

**Table 1:** The histone (de)methylation involved in osmolarity-related stress.

**Species**	**Component**	**Specificity**	**Stimuli**	**Target**	**Reference**
*Arabidopsis thaliana*	Unspecified	H3K4me3	dehydration	Unspecified	van Dijk et al., 2010
*Arabidopsis thaliana*	Unspecified	H3K4me3	light	*SOC1*	Aoki et al., 2019
*Arabidopsis thaliana*	ATX1	H3K4me3	dehydration	*NCED3*	Ding et al., 2011
*Arabidopsis thaliana*	Unspecified	H3K4me3	dehydration	*RD29B, RAB18*	Ding et al., 2012
*Arabidopsis thaliana*	Unspecified	H3K4me3	drought	*RD29A, RD29B, RD20, At2g20880*	Kim et al., 2008
*Arabidopsis thaliana*	Unspecified	H3K4me3	ABA	*MYB44*	Nguyen et al., 2019
*Arabidopsis thaliana*	ATX4, ATX5	H3K4me3	dehydration	*AHG3*	Liu et al., 2018b
*Arabidopsis thaliana*	Unspecified	H3K4me3	salt	*ABI1, ABI2, HAI1*	Nguyen et al., 2019
*Arabidopsis thaliana*	LHP1	H3K27me3	ABA	*ANAC019, ANAC055*	Ramirez-Prado et al., 2019
*Arabidopsis thaliana*	CAU1	H4R3sme2	Ca2+	*CAS*	Fu et al., 2013
*Arabidopsis thaliana*	CAU1	H4R3sme2	drought	*ANAC055*	Fu et al., 2017
*Arabidopsis thaliana*	JMJ17	H3K4me3	drought	*SnRK2.6*	Huang et al., 2019
*Arabidopsis thaliana*	JMJ30, JMJ32	H3K27me3	ABA	*SnRK2.8*	Wu et al., 2019a, 2019b
*Arabidopsis thaliana*	JMJ27	H3K9me2	drought	*GLOS2, RD20*	Wang et al., 2021a
*Arabidopsis thaliana*	JMJ17	H3K4me3	ABA	*ABI5*	Wang et al., 2021b
*Arabidopsis thaliana*	JMJ15	H3K4me2, H3K4me3	salt	*Unspecified*	Shen et al., 2014
*Arabidopsis thaliana*	LDL1, LDL2	Unspecified	ABA	*DOG1, ABA2, ABI3*	Zhao et al., 2015
*Arabidopsis thaliana*	Unknown	H3K27me3	salt priming	*Unspecified*	Sani et al., 2013
*Arabidopsis thaliana*	HDA6, HD2C	H3K9me2	ABA, salt	*ABI1, ABI2*	Luo et al., 2012
*Arabidopsis thaliana*	UBC1, UBC2	H3K4me3	salt	*MPK4, MYB42*	Sun et al., 2020
*Arabidopsis thaliana*	BPC	H3K27me3	unspecified	*ABI4*	Mu et al., 2017
*Arabidopsis thaliana*	VIL1	H3K27me3	ABA	*ABI3, ABI4, ABI5*	Zong et al., 2022
*Oryza sativa*	AGO2	H3K4me3, H3K27me3	salt	*OsBG3*	Yin et al., 2020
*Oryza sativa*	Unspecified	H3K4me3, H3K27me3	salt	*OsBZ8*	Paul et al., 2017
*Oryza sativa*	OsSDG708	H3K36me3	drought	*NCED3, NCED5*	Chen et al., 2021b
*Oryza sativa*	OsJMJ703	H3K4me3	drought	Unspecified	Song et al., 2018
*Oryza sativa*	Unspecified	H3K4me3	drought	Unspecified	Zong et al., 2013
*Oryza sativa*	OsJMJ710	H3K36me2	drought	*OsMYB48-1*	Zhao et al., 2022
*Zea mays*	Unknown	H3K4me2,H3K9me2,H3K27me2	salt, mannitol	Unspecified	Kamal et al., 2021
*Zea mays*	Unknown	H3K4m3,H3K36me3,H3K27m3	unspecified	natural antisense transcript genes	Xu et al., 2017
*Solanum lycopersicum*	Unknown	H3K9me2, H3K27me3	water deficit	*Asr2*	González et al., 2013
*Solanum lycopersicum*	SlJMJ4	H3K27me3	ABA	*SlABI5, SlNCED3, SlORE1, SlNAP2, SlSAG113, SlSAG12*	Ding et al., 2022
*Solanum lycopersicum*	SlSDG33, SlSDG34	H3K4me3, H3K36me3	drought	*SlRD29, SlERF3, SlTPL, SlRCD1, SlNAC064, SlPUB23, SlZAT10*	Zong et al., 2022
*Gossypium hirsutum*	AtHUB2	H2Bub1	drought	*DREB*	Chen et al., 2019
*Gossypium hirsutum*	GhJMJ34, GhJMJ40	Unspecified	salt	Unspecified	Sun et al., 2021
*Camellia sinensis*	CsSDG36	H3K4me2, H3K4me3	drought	Unspecified	Chen et al., 2021c
*Camellia sinensis*	Unspecified	H3K9me2	desiccation	Unspecified	Gu et al., 2021
*Triticum aestivum*	TaSDG1a − 7A, TaSDG16-3A, TaSDG22a-1D, TaSDG20-3D, TaSDG25c-5D, TaSDG51-2B	Unspecified	drought	Unspecified	Batra et al., 2020
*Dendrobium catenatum*	DcASHR3, DcSUVR3, DcATXR4, DcATXR5b, DcSDG49	Unspecified	drought-recovery	Unspecified	Chen et al., 2020
*Glycine max*	Unspecified	H3K27me3	salt	Unspecified	Sun et al., 2019
*Glycine max*	GmPHD6, GmLHP1	H3K4me0/1	salt	*CYP75B1, CYP82C4*	Wei et al., 2017
*Medicago sativa*	Unspecified	H3K4me3	salt	*MsMYB4*	Dong et al., 2020
*Ricinus communis*	Unspecified	H3K4me, H3K27me3	salt	*RSM1*	Han et al., 2020

This table presented part of but not all the existing reports on histone methylation with either direct or indirect relationship to osmotic stress.

## Two dynamic counterparts in stress-responsive gene transcription

### Histone methylation: Adding methyls for gene regulation

Osmotic stress and stress-triggered abscisic acid (ABA) signaling cast vast effects on the global landscape of histones. In the Arabidopsis genome, about 80% of the annotated genes bear H3K4me1, 84% bear H3K4me2 marks, and 62% bear H3K4me3 mark. In total, up to about 90% of annotated genes are methylated on H3K4 to different extent and a train of dehydration/ABA inducible genes marked by H3K4me3. Though the H3K4me3 peaks around the transcription start site (TSS) of most transcribed genes, dehydration and ABA can induce wider distribution of H3K4me3 profiles. Moreover, H3K4me1 and H3K4me2 are less sensitive to dehydration stress compared to H3K4me3 (van Dijk et al., 2010). In maize (*Zea mays*) seedlings stressed by salinity and drought, the active methylation mark H3K4me2 remarkably increases while the repressive methylation marks on H3K9 and H3K27 drop, implying a genome-wide gene activation through chromatin state might be involved in osmotic stress (Kamal et al., 2021). In soybean (*Glycine max*), salt stress induces *de novo* establishment of H3K27me3 to deactivate gene transcription (Sun et al., 2019). In the tea plants (*Camellia sinensis*), drought stress decreases the methylation of histone H3K4 (Chen et al., 2021c). These results pinpoint the correlation between stress-induced histone methylation and genome-wide transcription reprogramming.

Stress signals not only induce global changes in histone methylome but also specifically affects the methylation on stress-responsive genes. In Arabidopsis, *MYB44* is a highly active gene and a potent repressor of the salt-induced expression of *PROTEIN PHOSPHATASE TYPE2 C* (*PP2C*) genes. ABA lowers the nucleosome occupancy and mannitol induces a remarkable increase in H3K4me3 and H3ac both in the promoter and the gene body of *AtMYB44* (Nguyen and Cheong, 2018). The ABA-signaling components including *PP2C* genes (e.g., *ABI1*, *ABI2*, and *HAI1*) are upregulated under salt stress, accompanied with increased active markers H3ac and H3K4me3, and decreased nucleosome density. Under non-stress conditions, a functional SWI2/SNF2 chromatin remodeling ATPase BRAHMA (BRM) is required for the repression of *PP2C* genes (Nguyen et al., 2019). *NCED3* (*Nine Cis-Epoxycarotenoid Dioxygenase 3*) encodes a rate-limiting enzyme in the pathway of ABA biosynthesis and its transcription is fine-tuned to maintain proper stress response (Qin and Zeevaart, 1999; Iuchi et al., 2001; Tan et al., 2018). In response to osmotic stress, *NCED3* expression is dramatically upregulated and induces the ABA biosynthesis. In Arabidopsis, Drought decreases nucleosome occupancy in the *RD29A* (*RESPONSIVE TO DESICCATION 29A*) region, and H3K4me3 and H3K9ac deposition at *RD20* (*RESPONSIVE TO DESICCATION 20*) and *RD29A* to upregulate their expression (Kim et al., 2008). Arabidopsis TRX proteins (ARABIDOPSIS HOMOLOG OF TRITHORAX) including ATX1, ATX3, ATX4, and ATX5, are involved in deposition of H3K4me3 which is an active histone mark (de la Paz Sanchez et al., 2015; Pu and Sung, 2015; Chen et al., 2017; Song et al., 2021). ATX1 positively regulates the expression of *NCED3* through trimethylation of H3K4. The expression of *RD29A/B* is impaired in *atx1* mutants. Meanwhile, the stallment of RNA Polymerase II which is a core component of the transcription machinery mirrors the decrease of H3K4me3 (Ding et al., 2011, 2012). Collectively, ATX1 probably plays a multipurpose role in drought response through regulation of genes involved in both ABA biosynthesis and the signaling cascade. Other TRX members, ATX4 and ATX5 function redundantly in drought response. Following the perception of ABA signal, both ATX4 and ATX5 are directed to *AHG3 (ABA-HYPERSENSITIVEGERMINATION 3).* The association of ATX4 and ATX5 to *AHG3* locus increases H3K4me3 deposition and stallment of RNA polymerase II, leading to enhanced expression of *AHG3*, which finally decreases ABA-sensitivity (Liu et al., 2018b). Thus, ATX homologs might confer opposite stress response through shared biochemical activity but distinctive loci-specificity. These studies together illustrate a H3K4me3-based mechanism by which the ABA/drought response is fine-tuned through modulation of ABA-biosynthesis and ABA signaling (Ding et al., 2011; Liu et al., 2018b; Nguyen et al., 2019).

In addition to active marks including H3K4 methylation, repressive methylation marks on H3K27 and H4R3 also participate in osmotic stress. LHP1 (LIKE HETEROCHROMATIN PROTEIN 1), a component of PRC1 (POLYCOMB REPRESSIVE COMPLEX 1), mediates the H3K27me3 deposition on ABA-responsive genes to exert the transcription repression (Ramirez-Prado et al., 2019). CAU1(CALCIUM UNDERACCUMULATION 1), a H4R3sme2 methylase, binds to the *CAS (CALCIUM SENSING RECEPTOR)* promoter for H4R3sme2 methylation and consequently represses the expression of *CAS* to decrease stomatal closure and drought tolerance. However, this process is highly dynamic as elevated Ca^2+^ induces dissociation of CAU1 from the *CAS* promoter, further relieving the epigenetic repression effects (Fu et al., 2013). In another study, drought stress downregulates the expression of *CAU1*, which results in decreased H4R3sme2 in the promoter region and elevated expression of *ANAC055(ATAF-LIKE NAC DOMAIN CONTAINING PROTEIN 55)*. Upregulated *ANAC055* promotes proline accumulation through elevated expression of *P5CS1*(*DELTA1-PYRROLINE-5-CARBOXYLATE SYNTHASE 1*; Fu et al., 2017). These two studies have uncovered a negative role of H4R3 methylation in drought response through *CAS* for stomatal aperture and ANAC055 for proline decumulation.

There is mounting evidence that histone methylation is also an important regulatory mechanism of stress response in other plant species. The salt-responsive gene *OsBZ8* encoding a bZIP transcription factor bears distinctive H3K27me3 and H3K4me3 profiles both under resting conditions and stressful conditions, which might in part explain the different salt-tolerance across rice varieties (Paul et al., 2017). The rice ARGONAUTE2 (AGO2) is associated with *BIG GRAIN3(BG3)* locus and increase the H3K4me3 but decrease the H3K27me3. The activation of *BG3* transcription leads to enhanced salt response (Yin et al., 2020). *OsSDG708*, encoding a putative H3K36 methyltransferase, positively regulates drought tolerance by directly targeting and activating the crucial ABA biosynthesis genes *OsNCED3* and *OsNCED5* in rice. Overexpression of *OsSDG708* enhances the drought tolerance and increases the grain yield either, highlighting its potential role epigenetic regulation (Chen et al., 2021b). In dehydrated maize plants, higher active methylation (H3K36me3 and H3K4me3) and lower repressive methylation (H3K27me3) are deposited in the loci of antisense transcripts, indicating biogenesis of small RNAs might be regulated by histone methylation in drought response (Xu et al., 2017). In tomato (*Solanum lycopersicum*), two repressive histone methylation marks exhibit distinctive response to dehydration. The H3K27me3 barely changes while the H3K9me2 rapidly drops after dehydration (González et al., 2013). The pathogen- and drought-inducible *SlSDG33* and *SlSDG34*, which are homologous to Arabidopsis *SDG8*, negatively regulate drought tolerance by promoting the H3K4me3 and H3K36me3 deposition on a set of genes with negative regulatory roles in stress tolerance. *SlSDG33* and *SlSDG35* might act additively in drought response as the double mutant showed superior tolerance to the single mutants (Zong et al., 2022). During the postharvest desiccation process in tea plants, the ABA-biosynthesis genes are activated with elevated histone acetylation and decreased H3K9me2, leading to ABA accumulation (Gu et al., 2021). In salt-stressed castor beans (*Ricinus communis*), H3K4me3 and H3K27me3 showed a correlation with expression of the stress-regulated genes. RSM1, a MYB-like transcription factor, was under dynamic bivalent control of H3K4me3 (active) and H3K27me3 (repressive) by salt stress (Han et al., 2020). In bread wheat (*Triticum aestivum* L.), 166 SDG members are identified, 36 of which had incomplete SET domain. During the seedling stage, 30 out of the 166 *TaSDG*s are downregulated by heat and drought, suggesting they are involved in stress response (Batra et al., 2020). In a Chinese medicinal monocot, the *Dendrobium catenatum*, 5 out of 42 *SDG* genes showed altered expression level during drought-recovery (Chen et al., 2020). Taken together, the histone methylation in osmotic stress responses might be a common regulatory mechanism across the plant species.

All the eukaryotic cells have similar chromatin structure which is subject to a set of shared epigenetic modifications. It is reasonable that those modifications are likely conserved to some extent. For instance, ectopic expression of *AtHUB2*, encoding an Arabidopsis histone mono-ubiquitination E3 ligase, significantly improves the yield of transgenic cotton (*Gossypium hirsutum*) plants under drought stress conditions and concomitantly enhances the drought tolerance. By contrast, RNAi knockdown of *GhHUB2* genes decreased the drought resistance. The ectopically expressed AtHUB2 directly interacts with the endogenous GhH2B to deposit H2Bub1 at a stress-responsive gene *GhDREB (DEHYDRATION-RESPONSIVE ELEMENT BINDING PROTEIN)*, resulting in increased H3K4me3 and gene activation (Chen et al., 2019). Considering the cross-species activity of AtHUB2, the role of H2Bub1 in drought tolerance is conserved in Arabidopsis and cotton plants. Moreover, this epigenetic regulation has promising future as the agronomic traits of transgenic cotton under drought condition were dramatically improved. Ectopic expression of *CsSDG36* from the tea plants, which is homologous to *ATX4* of Arabidopsis, downregulates stomatal development-related genes, increases stomatal density, and consequently decreases drought tolerance in Arabidopsis (Chen et al., 2021c). As aforementioned, both genes involved in stress-signaling and catalytic enzymes of stress signaling are subject to various histone methylations (Ding et al., 2011; Liu et al., 2018b; Nguyen et al., 2019). Though there are extensive studies revealing the role of histone methylation, their regulatory targets seem to be unpredictable based on the existing literature (as summarized in Table 1). Such phenomenon is likely due to variations in the genomic context and different evolutionary trajectories across the species. Meanwhile, all the epigenetic codes must have their genetic basis which is encoded by the DNA sequence. Development of new methodologies in high-throughput sequencing and advances in bio-informatics of learning the genome-epigenome-phenome relationship will greatly benefit epigenetic studies in crops as well as provide insights into the nucleic acid foundation of histone codes (Li et al., 2022).

### Histone demethylation: Erasing the histone marks for more plasticity

Since the aforementioned histone methylation can either be active or repressive, the demethylation also plays different roles in gene transcription activity depending on their target sites and the chromatin context. The SnRKs (SNF1-RELATED PROTEIN KINASE) are important signaling component of osmotic stress. In Arabidopsis, H3K4me3 in the regions of promoter and gene body of *SnRK2.6* can be demethylated by JMJ17 (Jumonji Domain-Containing Protein 17). Functional loss of *JMJ17* leads to hyper-expression of *SnRK2.6*, increased ABA-sensitivity, reduced stomatal aperture, and enhanced the tolerance to drought stress (Huang et al., 2019). ABI3 activates the transcription of *JMJ30* at post-germination stage. Then *JMJ32* and the upregulated *JMJ30* together control the ABA-mediated post-germination growth arrest through H3K27me3 demethylation and activation of *SnRK2.8* (Wu et al., 2019a,b). JMJ15 preferentially represses the genes marked by H3K4me2 and H3K4me3 probably through histone demethylation and enhances salt tolerance (Shen et al., 2014). Hyper-expression of *JMJ15* down-regulates a consortium of stress-responsive genes encoding transcription factors including *ZAT10*, *WRKY33*, *WRKY44*, *CBF2, ERF6*, and *ERF 10*. Meanwhile, the over-presented function of JMJ15 leads to higher expression level of *RD29A*, *RD22*, and *COR15*. However, this study failed to identify the direct targets of JMJ15, which most likely underpin the salt tolerance. Arabidopsis histone demethylases LDL1 and LDL2 (LYSINE-SPECIFIC DEMETHYLASE LIKE 1 and 2) act redundantly in repressing the seed dormancy, and their function is genetically dependent on seed-specific gene *DOG1 (DELAY OF GERMINATION1)*, ABA-biosynthesis gene *ABA2 (ABA DEFICIENT 2)* and ABA-signaling gene *ABI3* (*ABA-INSENSITIVE3;* Zhao et al., 2015). Thus, *DOG1*, *ABA2*, and *ABI3* are potential targets of LDL1/2.

In non-model plants, there is mounting evidence of histone demethylation involved in osmotic stress. OsJMJ710 targets to *MYB48-1* and demethylates H3K36me2, and dowregulates the expression of *MYB48-1*, leading to repressed drought tolerance in rice (Zhao et al., 2022). OsJMJ703 negatively regulates both the floral development and the drought tolerance. Though its targets have not been identified, its potential role as a demethylase of H3K4me3 is proposed due to the observation of increased global H3K4me3 in knock-down transgenic lines (Song et al., 2018). Tomato SlJMJ4 positively regulates dark- and ABA-induced leaf senescence by two partially overlapping mechanisms. In dark, SlJMJ4 is directed to the loci of *SlORE1*, *SlNAP2*, *SlSAG113*, and *SlSAG12*. In response to ABA, SlJMJ4 is targeted to *SlABI5* and *SlNCED3*, in addition to *SlORE1*, *SlNAP2*, *SlSAG113*, *SlSAG12*. SlJMJ4 activates the transcription through removal of H3K27me3 in both processes (Ding et al., 2022). Considering the importance of *ABI5* in ABA signaling and *NCED3* in ABA biosynthesis, SlJMJ4 might also be involved in regulation of ABA-dependent drought tolerance. Moreover, there is masssive evidence showing that histone demethylation is responsive to osmotic stress though the mechanism has not been fully specified. Pan-genomic analysis in 11 rice species has identified 151 JmjC genes which suggests JmjC family underwent duplication and diversification during evolution. Some of the rice JmjC genes are involved in response to salt (Chowrasia et al., 2018). There are 64 JmjC genes in the genome of allotetraploid *Brassica napus*, of which 29 are from *Brassica rapa* and 23 from the *Brassica oleracea*. The *BnaKDM5* (*lysine demethylase 5*) subfamily genes respond to stresses including salt, drought and high temperature (He et al., 2021). In cotton species, JmjC genes are divided into five subfamilies. *Gossypium raimondii* has 25 JmjC genes, *Gossypium arboreum* has 26, *Gossypium hirsutum* has 52, and *Gossypium barbadense* has 53. Several GhJMJs respond to salt and PEG treatment. Ectopic expression of GhJMJ34/40 imparts osmotic tolerance *Saccharomyces cerevisiae* (Sun et al., 2021). In birch, a total of 21 JmjC domain-containing histone demethylase proteins (JHDMs) are identified and classified into five subfamilies. In their promoter regions, cis-elements associated with hormone and abiotic stress responses are overrepresented. Their expression profiles also support their role in abiotic stress (Chen et al., 2021a). Thus, the genes encoding histone demethylases themselves are regulated by osmotic stress. In conclusion, JMJ- or LSD1-mediated histone demethylation allows regulatory plasticity of gene transcription under stress.

## Cross-talks of histone (de)methylation in response to osmotic stress

### The trithorax proteins

Histone methylation extensively cross-talks with other mechanisms both directly and indirectly to orchestrate the transcription of stress-related genes. Trithorax (TRX) group proteins are evolutionarily conserved activators with putative methylation activity on H3K4. The Arabidopsis TRX group consists of 12 members including ATX1–5 and ATX-RELATED (ATXR) 1–7 in (Alvarez-Venegas and Avramova, 2002; Veerappan et al., 2008; Chen et al., 2017). ATX5 deposits H3K4me3 at *HY1* locus, activating its transcription to represses the transcription of *ABI4 (ABA INSENSITIVE4)*. By contrast, glucose deactivates ATX5 thus diminishing H3K4me3 on *HY1* locus, which further leads to a mitigation of HY1-repressed *ABI4* expression (Liu et al., 2018a). The ATX1-HY1-ABI4 module epigenetically couples glucose signaling with transcription. Since the ABI4 is an important ABA-signal transducer in plants, this module might also be involved in ABA-signaling. Stomatal movement plays a critical role in drought tolerance as the stomata controls the transpiration rate. Light induces stomatal opening through H3K4me3 deposition at *SOC1(SUPPRESSOR OF OVEREXPRESSION OF CO 1)* gene, which is dependent of *FT* (*FLOWERING LOCUS T;* Aoki et al., 2019). Therefore, H3K4me3 might integrate light signaling into flowering and drought response.

### The ASH proteins

Though Arabidopsis SDG26 (SET DOMAIN GROUP 26) which belongs to the ASH family has been characterized as a histone methyltranseferase with H3K36 specificity, which marks gene activation (Berr et al., 2015). Paradoxically, loss of functional FLD/LD/SDG26 leads to over-accumulation of H3K4me1/2 and H3K36me3, which hints SDG26 might be a versatile modifier involved both in gene activation and repression (Fang et al., 2020). Later in another study, the authors found that FLD/SDG26/LD interacts with R-loop to slow both the transcription initiation and the elongation (Xu et al., 2021). Since the ASH-group HKMTs are involved in drought tolerance (Chen et al., 2021b), the output when multiple epigenetic mechanisms are combinatorially involved needs to be reconsidered.

### The polycomb-group proteins

The PcG proteins and TrxG proteins represent two groups of histone modifiers with contrasting roles in gene transcription. PcG proteins maintain gene repression through deposition of repressive histone marks including H3K27me3 and H2Aub1 while TrxG proteins can function oppositely by catalyzing active histone marks such as H3K4me3. A well-characterized antagonistic model is the PcG-TxG counteraction in flowering. However, the ATX1 (TrxG)-ULT1 (TrxG)-EMF1 (PcG) module can also function synergistically and additively during seed germination (Xu et al., 2018). Moreover, a novel hypothesis was raised that PcG and TrxG complexes could function in concert to deposit bivalent chromatin marks as the H3K27me3 and H3K4me3 level in *clf* and *atx1* double mutant was partially restored at the silent *AG* locus (Saleh et al., 2007). There is crosstalk in different PcG groups. Generally, Histone ubiquitination by PRC1 requires H3K27me3 deposited by PRC2 in Arabidopsis. On the contrary, PRC1 seems to function independently of PRC2 and is required for PRC2’s function in other reports (Merini and Calonje, 2015; Zhou et al., 2017; Wang and Shen, 2018). Histone ubiquitination also participates in interplay with histone methylation. In Arabidopsis, UBC1/2 (UBIQUITIN CARRIER 1/2) encode two E2 conjugated enzymes which are invoved in Histone H2B monoubiquitination (H2Bub1). Both UBC1 and UBC2 positively regulate salt response through the MPK4-MYB42-SOS2 module. H2B monoubiquitination mediated by UBC1/2 enhances H3K4me3 at both *MYB42* and *MPK4* and reinforces their expression (Sun et al., 2020).

### The histone readers

The histone modifiers are also recruited to specific genomic loci through assembly into protein complexes containing DNA-binding subunits. The PhD-finger protein VIL1 (VIN3-LIKE1) can directly target to *ABI3*, *ABI4*, and *ABI5*, which encodes important transcription factors required for ABA signaling. Further, VIL1 recruits PRC2 complex to trimethylate H3K27me3 and repress the transcription of ABI3/4/5. Loss-of-VIL1 leads to hypersensitivity to ABA and enhanced drought tolerance (Zong et al., 2022). GmPHD6 (PLANT HOMEODOMAIN 6) interacts with the GmLHP1/2 coactivators through the PHD domain to form a transcriptional activation complex. GmPHD6 recognizes the G-rich elements in target gene promoters and is recruited by non-methylated (H3K4me0) and low methylated histone (H3K4me1,2) but not high methylated histone (H3K4me3). Once recruited, GmPHD6 further recruits GmLHP1 to activate ABA-responsive genes including *CYP75B1* and *CYP82C4*. Therefore, GmPHD6 improves the salt tolerance in a GmLHP1-dependent manner (Wei et al., 2017). In most studies, LHP1 has been proposed as a subunit of PRC1 (Polycomb Repressive Complex 1) to repress gene transcription through mono-ubiquitination of H2A (Xu and Shen, 2008; Bratzel et al., 2010; Wang et al., 2014), and an accessory protein of PRC2 (Polycomb Repressive Complex2) to repress gene transcription through H3K27me3 (Zhang et al., 2007; Yuan et al., 2016). Therefore, contradiction of how GmLHP1/2 and GmPHD6 are coordinated to activate gene transcription is yet to be addressed. On the other hand, the plant homeodomain (PHD) finger proteins can recognize and bind to modified histone H3 and function as histone code readers (Shi et al., 2007; Bürglin and Affolter, 2016; Wei et al., 2017), the recognition of existing histone modification might also be instrumental to subsequent modifications for further gene regulation in response to salt stress. Besides the existing histone modifications, the cis-elements on the genome also bear important information for histone modifications. Such is the case that the cis-elements in the genic region of *MsMYB4*, are required for its expression and the local H3K4me3 and H3K9ac induced by salt in alfafa (*Medicago sativa;* Dong et al., 2020). In another way, some chromatin-associated factors can modulate histone methylation through physical interaction with histone modifiers. BPC (BASIC PENTACYSTEINE) proteins binds to *ABI4* promoter and physically recruit the PRC2 to deposit H3K27me3, further leading to repressed *ABI4* expression (Mu et al., 2017). Histone readers VP1/ABI3-LIKE 1/2 can recognize a cis-regulatory element at the *FLC* locus and recruits HDA9 for locus-specific H3K27 deacetylation. Deacetylated H3K27 in the *FLC* region is subsequently marked with H3K27me3 by PRC2 (Zeng et al., 2019). This hierarchy between histone deacetylation and H3K27me3 might enable plants to flower smartly in acclimation the environment because the HDA9 is involved in various stress response (Chen and Wu, 2010; Zheng et al., 2016, 2020; Shen et al., 2019; Van Der Woude et al., 2019; Baek et al., 2020; De Rooij et al., 2020). Other histone deacetylases including HD2C, HDA6 and HDA19 can regulate the histone methylation at the ABA-responsive genes including *ABI1* and *ABI2* (Luo et al., 2012).

### The non-coding RNAs

The noncoding RNAs (ncRNAs) are functional ribonucleic acids that do not encode proteins or function independently of their peptide products. The role of noncoding RNAs including miRNA and lncRNAs in mediating the chromatin state has revealed (Böhmdorfer and Wierzbicki, 2015). Hereby, the histone methylation mediated by non-coding RNAs is discussed.

microRNAs (miRNAs) play an important role in plant stress responses. The Arabidopsis *miR778* targets to the *SUVH6* which encoding a histone H3K9 methyltransferase. The downregulation of SUVH6 mediated by *miR778* causes dramatic upregulation of Pi deficiency-responsive genes. Though the direct evidence of H3K9 methylation at these loci is missing, it is likely that *miR778* regulates the Pi-deficiency responsive genes transcription through regulation of SUVH6-mediated H3K9 methylation (Wang et al., 2015b). In a recent study, the *miR778* negatively regulate both SUVH5 and SUVH6, thus activating the nematode-responsive genes (Bennett et al., 2022). These two studies support the multifaceted role of *miR778* both in abiotic and biotic stress by targeting the histone methyltransferase genes to reduce the deposition of H3K9 methylation. However, the role of miRNAs in osmotic stress-induced histone (de)methylation remains elusive.

The lncRNAs probably serve both shared and distinctive roles the in plant stress responses (Di et al., 2014). The expression of *DROUGHT INDUCED lncRNA (DRIR)* can be dramatically upregulated. Hyper-expression of *DRIR* increases tolerance to drought and salt stress in an ABA-dependent manner. A consortium of genes involved in ABA signaling, water transport, and other stress response are misregulated by hyperexpression of *DRIR* (Qin et al., 2017). lncRNAs regulate histone methylation by recruiting histone modifiers to regulate gene transcription. *COOLAIR* and *COOLAIR* are two distinct lncRNAs transcribed from the 3′ end and the first intron of *FLC*. *COOLAIR* recruits CLF which is a component of PRC2 to *FLC* to establish H3K27me3 for gene repression (Heo and Sung, 2011). The *COOLAIR* antisense transcripts are targeted to *FLC* locus. The biogenesis of *COOLAIR* transcripts precedes the deposition of H3K27me3 at *FLC* locus by prolonged cold, which mediated the replacement of H3K36 methylation with H3K27me3 in the intragenic region. Interestingly, the *COOLAIR* lncRNA mediates a cold-induced synchronization from H3K36 methylation to H3K27 methylation, thus repressing the transcriptional activity of *FLC*. In the study, the researchers proposed that *COOLAIR* worked independently of the polycomb complex (Csorba et al., 2014). Likewise, *MAS4* is the antisense transcript of *MAF4* (*MADS AFFECTING FLOWERING4*). *MAS4* is associated with WDR5a and recruits it to *MAF4* locus, which further promotes the H3K4me3 and activate the transcription of *MAF4* (Zhao et al., 2018). A short splice variant of *COOLAIR* affects the recruitment of the histone demethylase FLD (FLOWERING LOCUS D) to *FLC*, leading to demethylation of H3K4 at *FLC* (Liu et al., 2007; Marquardt et al., 2014). The *FLC* gene is a hot topic and represents a recognized platform to study epigenetic basis in the model plants (Whittaker and Dean, 2017). However, the direct evidence of lncRNA-regulated histone methylation in osmotic response is still missing. In other model species, recent studies might better our understanding of the relationship between lncRNA and histone methylation as the epigenetic basis is often highly conserved in eukaryotes. In mice, transcription factor OCT4 activates the expression of lncRNA *Suv39h1as* which targets to the *Suv39h1* and down regulates its expression. As the *Suv39h1* is a major methyltransferase catalyzing the di- and tri-methylation of H3K9, *Suv39h1as* implements negative regulation on the H3K9me2 and H3K9me during the differentiation of embryonic cells. Thus, the lncRNA-regulated can also be indirect (Bernard et al., 2022).

### The histone demethylases

Similar to the interactive networks entailing histone methylation, the cross-talks sponsored by histone demethylation also play an unneglectable role in response to osmotic stress. In *Arabidopsis thaliana*, WRKY40 recruits histone demethylase JMJ17 to the *ABI5* chromatin to deplete the deposition of H3K4me3. The decreased H3K4me3 level consequently deactivates the transcription of *ABI5*. Meanwhile, WRKY40 represses the transcription of *HY5* to downregulate *ABI5* expression. Upon stress, WRKY40 and JMJ17 are dissociated from *ABI5* loci to withhold the transcriptional repression. The elevated ABI5 protein forms heteromeric dimer with HY5, and reinforces its own expression. Thus under resting conditions, this mechanism forms a double-safety clutch to repress constitutive ABA-response. Under stressful conditions, the concomitant release of WRKY40-JMJ17 and WRKY40-HY5 can boost the expression of *ABI5* by increasing both the chromatin accessibility and activity of transcription factor (Wang et al., 2021b). This model represents a multilayer crosstalk between transcription factors and histone methylation status. Under non-stressful conditions, RPN1a (REGULATORY PARTICLE NON-ATPASE 1a) interacts with JMJ27 and mediates its degradation through the 26S proteasome pathway. Drought stress diminishes RPN1a abundance and indirectly elevates the protein level of JMJ27 to reinforce the activation of *GOLS2* and *RD20* through demethylation of H3K9me2 (Wang et al., 2021a). The proteasome-mediate turnover of histone demethylases provides another layer of regulation *via* the enzyme homeostasis. The enzymatic activity of JmjC proteins requires cofactors including ferrous iron and alpha-ketoglutarate in the oxidative demethylation reaction. Thus, it is convincing that disruption in the biochemical pathway required for demethylation can interfere with the demethylation process. Blocking the alpha-ketoglutarate biogenesis pathway concomitantly impairs the function of JMJ14, JMJ15, and JMJ18, leading to a global increase in H3K4me3 which further affects the thermosensory response (Cui et al., 2021). This results represent a metabolic regulation of histone methylation. An antagonistic model has been proposed where the PRC2 complex deposits H3K27me3 to repress gene transcription but JmjC proteins erase methylation from H3K27me3 to activate gene transcription (Crevillén, 2020). It is noteworthy that plant histone demethylases counteract the gene repression through erasure of the repressive histone methyl marks in most reported cases, which establish a de-repression mechanism upon stress.

## Histone methylation and stress memory

Though a multitude of evidence show that histone methylation status is closely associated with the gene transcription activity, there are some exceptions. For instance, H3K4me3 enrichment on 4,837 rice genes in the genome were altered by drought stress while only a small proportion of them showed altered mRNA level (Zong et al., 2013), hinting a stress-memory imposed by drought through H3K4me3. In Arabidopsis, previous transient hyperosmotic stress imposes a long somatic memory by shaping the epigenomic landscape. Stress-induced H3K27me3 seems to be stable at least for 10 days before retreated to the normal level. By contrast, the H3K4me2, H3K4me3, and H3K9me2 islands are barely changed (Sani et al., 2013). The methylation on H3K4 might persist during stress-recovery stage (Ding et al., 2011, 2012). Likewise, H3K4me3 mediated by SDG25 and ATX1 plays an important role in heat-responsive gene expression, and is required for establishment of the heat stress memory (Song et al., 2021). However, functional loss of the H3K4me3 catalyzer ATX1 has no obvious impact on drought stress-memory in Arabidopsis (Ding et al., 2012). The genic loci of some drought-responsive transcription factors are less enriched in H3K27me3 in Arabidopsis, which might contribute to more efficient response to recurring drought (Ramirez-Prado et al., 2019). JMJ-mediated demethylation of H3K27me3 plays an indispensable role in heat acclimation with an imprinted stress memory at *HSP22* and *HSP17.6C* loci, which potentiates their expression when heat stress reoccurs (Yamaguchi et al., 2021). These studies have suggested that different memory mechanism might be involved depending on the stress type. *APX2* (*ASCORBATE PEROXIDASE2*) gene shows stress memory after heat stress, correlating with persisting transcription and H3K4 hyper-methylation. Using a heat-inducible dCas9 to target a JMJ demethylase domain required for methylation of H3K4 significantly reduces the stress-induced H3K4me3 and swipes off the transcriptional memory (Oberkofler and Bäurle, 2022). Collectively, stress-memory established by histone methylation is possibly a common mechanism allowing plants to combat the environmental challenges (Ding et al., 2012; Sani et al., 2013; Kinoshita and Seki, 2014; Avramova, 2015; Liu et al., 2018b; Zhang et al., 2020; Song et al., 2021; Yamaguchi et al., 2021). Moreover, editing the epigenome might not only be a powerful tool for studying chromatin basis of stress memory but also at the frontline of utilization of epigenetics in field crops.

## Conclusion and future perspectives

**Figure 2:**
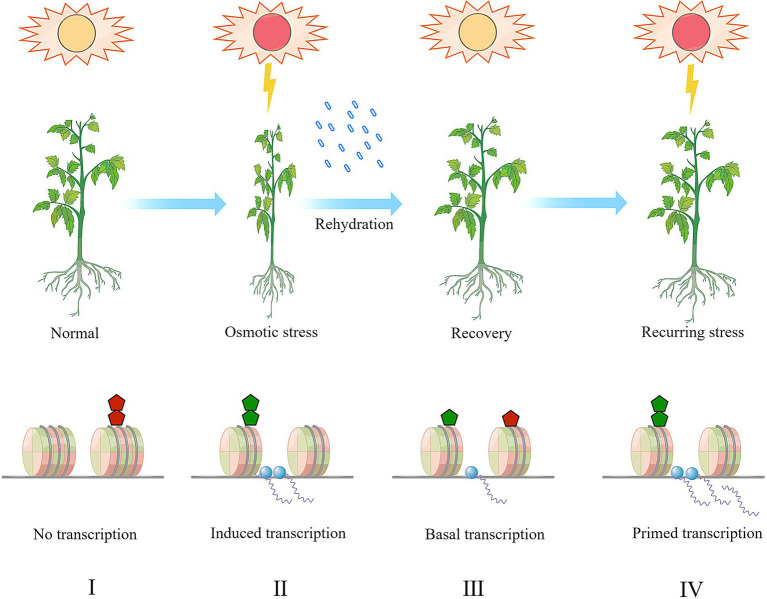
A hypothetic model of stress-memory and histone (de)methylation in plants combating recurring osmotic stress. The histone methylation dynamics at stress responsive genes and the transcriptional activation was exemplified and presented in this model. Under normal condition (I), repressive histone methylation (the red pentagon, e.g., H3K9me1/2/3, H3K27me1/2/3) is enriched and the chromatin is inaccessible to the transcription machinery (the blue dots). The low accessibility of chromatin results in transcriptional repression. Upon a primary drought response (II), the enrichment of repressive histone methylation is depleted the and active histone methylation (the green pentagon, e.g., H3K4me1/2/3, H3K36me1/2/3) is deposited to form an open chromatin status. Correspondingly, the stress responsive genes are transcribed upon induction. After recovery (III), the histone methylation pattern persists for a certain period and then both the active and repressive methylation marks gradually retreat to the normal level. The stress memory is imprinted during the transition from the stage (II) to the stage (III). During this stage, the transcription activity might be maintained in a lower basal level. When a secondary stress occurs (IV), the plants combat the stress more efficiently as the stress-responsive genes have been primed, and the active histone methylation and stallment of transcription machinery allow plants with quicker or more robust response, and finally reduces the plant vulnerability to stress. During the stage (IV), the stress memory stored on the histones is re-accessed and translated into gene transcription activity.

The *trans-cis* interaction between transcription factors and target DNA sequences has been well documented and the input of stress signals determines the output of gene transcription. Nonetheless, there remains gaps between the gene transcription and the anchoring of transcription factors, wherein chromatin accessibility might play a critical role. Unlike DNA methylation or histone acetylation, which marks gene repression and activation respectively, histone methylation is one of the most functionally diversified epigenetic mechanism as it can either exert activation or repression on target genes depending on the site of lysine residues. Moreover, the histone lysine can be methylated to different extent enabling plasticity in gene regulation. Here in this review, recent advances in the histone methylation and demethylation which establish histone methylome and orchestrate gene transcription in response to osmotic stress have been summarized and discussed. Apart from the conserved biochemical activity of histone methylation writers and erasers, their specificity is yet to be elucidated. Another unneglectable aspect is the cross-talks between histone methylation and other regulatory mechanisms including histone acetylation, DNA methylation, histone ubiquitination, transcription factors and small RNAs, etc. In some cases, well-recognized antagonistic regulators can work synergistically, which further hinders our in-depth understanding of the role of loci-specific histone methylation. Though the complexity of histone methylation and gene transcription has not been fully detangled in lab, the potential of histone methylation in field comes into sight as a set of studies on stress memory (also known as “priming”) have revealed that stress can impose both somatic retention and transgenerational inheritance accompanied with multiple epigenetic modifications, avoiding growth penalty caused by constitutive expression of stress response (Figure 2). Conclusively, we propose three directions as follows, toward which the study on histone methylation might be greatly advanced:

1. The loci-specificity of histone methylation;
2. The interplay and hierarchy between histone methylation and other mechanisms;
3. The formation, maintenance, and erasure of histone methylation-based stress memory.

## Author contributions

MX conceived this review and drafted the manuscript. JW participated in the figure configuration. FX conducted this work and gave insightful viewpoints to the manuscript. All authors contributed to the article and approved the submitted version.

## Funding

The work was supported by Taishan Scholars program of Shandong Province (tsqn201812018), Natural Science Foundation of Shandong Province (ZR2019ZD16), and Project for Scientific Research Innovation Team of Young Scholar in Colleges and Universities of Shandong Province (2020KJE002).

## Conflict of interest

The authors declare that the research was conducted in the absence of any commercial or financial relationships that could be construed as a potential conflict of interest.

## Publisher’s note

All claims expressed in this article are solely those of the authors and do not necessarily represent those of their affiliated organizations, or those of the publisher, the editors and the reviewers. Any product that may be evaluated in this article, or claim that may be made by its manufacturer, is not guaranteed or endorsed by the publisher.
